# Formulation and Application of Optimal Homotopty Asymptotic Method to Coupled Differential - Difference Equations

**DOI:** 10.1371/journal.pone.0120127

**Published:** 2015-04-14

**Authors:** Hakeem Ullah, Saeed Islam, Ilyas Khan, Sharidan Shafie, Mehreen Fiza

**Affiliations:** 1 Department of Mathematics, Abdul Wali Khan University Mardan, 23200 Pakistan; 2 College of Engineering Majmaah University, Majmaah, Saudi Arabia; 3 Department of Mathematical Sciences, Faculty of Science Universiti Teknologi Malayisa (UTM) 81310 Skudai, Johar Bohru, Johor, Malaysia; University of Minnesota, UNITED STATES

## Abstract

In this paper we applied a new analytic approximate technique Optimal Homotopy Asymptotic Method (OHAM) for treatment of coupled differential- difference equations (DDEs). To see the efficiency and reliability of the method, we consider Relativistic Toda coupled nonlinear differential-difference equation. It provides us a convenient way to control the convergence of approximate solutions when it is compared with other methods of solution found in the literature. The obtained solutions show that OHAM is effective, simpler, easier and explicit.

## Introduction

In physical and nonlinear science the DDEs play a vital role in modeling of the complex physical phenomena. The DDEs models are used in vibration of particles in lattices, the flow of current in a network, nonlinear fiber arrays, energy transfer in harmonic crystals, nonlinear charge, excitation transport in biological macromolecules and the pulses in biological chains. The models containing DDEs have been investigated by numerical techniques such as discretizations in solid state physics and quantum mechanics. In the last decade’s most of the research work has been done on DDEs. Yamilov et al. [1, 2] attributes their work to classification of DDEs and connection of integrable partial differential equation (PDEs) and DDEs [3, 4]. In [5–8] the exact solutions of the DDEs have been studied. For the solution of the DDEs L. Zou et. al. extended the Homotopy Analysis Method (HAM) [9–10]. Z. Wang et. al. extended Adomian Decomposition Method (ADM) for solving nonlinear Differnece-Diffrential equations (NDDEs) and got a good accuracy with the analytic solution [11]. The Adomian Decomposition Method (ADM) has been used by M.A. Abdou for the solution of Relativistic Toda coupled nonlinear differential-difference equation [12]. Recently, Vasile Marinca et al. introduced Optimal Homotopy Asymptotic Method (OHAM) [13–17] for the solution of nonlinear problems. The validity of OHAM is independent of whether or not the nonlinear problems contain small parameters.

The motivation of this paper is to extend OHAM for the solution of nonlinear coupled differential-difference equations (NCDDEs). In [17–19] OHAM has been proved to be useful for obtaining an approximate solution of nonlinear boundary value problems by M. Idrees et al. H. Ullah et al. have extended and applied OHAM to a system of nonlinear boundary value problems [20–25]. In this work, we have proved that OHAM is also useful and reliable for the solution of NCDDEs showing its validity and great potential for the solution of NCDDEs phenomenon in science and engineering.

The paper has been organized as follow. In the following section (Basic Mathematical Theory of Extended OHAM [Eqs 1–18]), the Formulation of OHAM for the NCDDEs is given an Relativistic Toda coupled nonlinear differential-difference equation. In the subsequent section (Application of Modified OHAM to Coupled Differential-Difference Equations [Eqs 19–35]), the effectiveness of OHAM formulation for NCDDEs has been studied.

## Basic Mathematical Theory of Extended OHAM

Let us take OHAM to the following differential-difference equation

$$\begin{array}{cc} \begin{array}{c} 𝒜\left(u_{n}\left(t\right),v_{n}\left(t\right)\right)+g\left(t\right)=0,\;\\ 𝒜\left(v_{n}\left(t\right),u_{n}\left(t\right)\right)+f\left(t\right)=0, \end{array} & n\in \Omega \end{array}$$ (1)

with boundary conditions

$$\begin{array}{cc} \begin{array}{c} 𝓑\left(u_{n}\left(t\right),\frac{\partial u_{n}\left(t\right)}{\partial t}\right)=0,\\ 𝓑\left(v_{n}\left(t\right),\frac{\partial v_{n}\left(t\right)}{\partial t}\right)=0. \end{array} & n\in \Gamma \end{array}$$ (2)

Where *A* is a differential operator, *u*_*n*_(*t*), *ν*_*n*_(*t*) is an unknown function, *n* and *t* denote spatial and temporal independent variables, respectively, Γ is the boundary of Ω and *f*(*t*), *g*(*t*) is a known analytic function. *A* can be divided into two parts *L* and *N* such that

𝒜=𝓛+𝓝 (3)

*L* is the simpler part of the partial differential equation which is easier to solve, and *N* contains the remaining part of *A*. So Eq 1 can be written as

$$\begin{array}{l} 𝓛\left(u_{n}\left(t\right)\right)+g\left(t\right)+𝓝\left(u_{n}\left(t\right),u_{n-k}\left(t\right),u_{n+k}\left(t\right),v_{n}\left(t\right),v_{n-k}\left(t\right),v_{n+k}\left(t\right)\right)=0,\\ 𝓛\left(v_{n}\left(t\right)\right)+f\left(t\right)+𝓝\left(v_{n}\left(t\right),v_{n-k}\left(t\right),v_{n+k}\left(t\right),u_{n}\left(t\right),u_{n-k}\left(t\right),u_{n+k}\left(t\right)\right)=0, \end{array}$$ (4)

According to OHAM, one can construct an optimal homotopy *ϕ*_*n*_(*t*, *q*): Ω×[0, 1] →ℜ, *φ*_*n*_(*t*, *q*) Ω×[0, 1] →ℜ satisfying

$$\begin{array}{l} \left(1-q\right)\left[𝓛\left(\phi_{n}\left(t,q\right)\right)+g\left(t\right)\right]=H_{1}\left(q\right)\left[\begin{array}{l} 𝓛\left(\phi_{n}\left(t,q\right)\right)+g\left(t\right)+\\ 𝓝\left(\phi_{n}\left(t,q\right),\phi_{n-k}\left(t,q\right),\phi_{n+k}\left(t,q\right)\right) \end{array}\right],\\ \left(1-q\right)\left[𝓛\left(\varphi_{n}\left(t,q\right)\right)+f\left(t\right)\right]=H_{2}\left(q\right)\left[\begin{array}{l} 𝓛\left(\varphi_{n}\left(t,q\right)\right)+f\left(t\right)+\\ 𝓝\left(\varphi_{n}\left(t,q\right),\varphi_{n-k}\left(t,q\right),\varphi_{n+k}\left(t,q\right)\right) \end{array}\right], \end{array}$$ (5)

$$\begin{array}{l} 𝓑\left(\phi_{n}\left(t,q\right),\frac{\partial \phi_{n}\left(t,q\right)}{\partial t}\right)=0,\\ 𝓑\left(\varphi_{n}\left(t,q\right),\frac{\partial \varphi_{n}\left(t,q\right)}{\partial t}\right)=0 \end{array}$$ (6)

where *q*∈[0, 1] is an embedding parameter, *ϕ*_*n*_(*t*, *q*), *φ*_*n*_(*t*, *q*) is an unknown function, *H*_1_(*q*), *H*_2_(*q*) is a nonzero auxiliary function. The auxiliary function *H*_1_(*q*), *H*_2_(*q*) is nonzero for *q* ≠ 0 and *H*_1_(0) = 0 = *H*_2_(0). Eq 6 is the structure of OHAM homotopy. Clearly we have

$$\begin{array}{l} q=0\Rightarrow H_{1}\left(\phi_{n}\left(t;0\right),0\right)=𝓛(\phi_{n}(t;0))+g(t)=0,\\ q=0\Rightarrow H_{2}\left(\varphi_{n}\left(t;0\right),0\right)=𝓛(\varphi_{n}(t;0))+f(t)=0,\\ q=1\Rightarrow H_{1}\left(\phi_{n}\left(t;1\right),1\right)=H_{1}(1)\left\{𝒜(\phi_{n}(t;1))+g(t)\right\}=0,\\ q=1\Rightarrow H\left(\varphi_{n}\left(t;1\right),1\right)=H_{2}(1)\left\{𝒜(\varphi_{n}(t;1))+f(t)\right\}=0. \end{array}$$ (7)

Obviously, when *q* = 0 and *q* = 1 we obtain

$$\begin{array}{l} q=0\Rightarrow \phi_{n}(t,0)=u_{n0}\left(t\right),\\ q=0\Rightarrow \varphi_{n}(t,0)=v_{n0}\left(t\right),\\ q=1\Rightarrow \phi_{n}\left(t,1\right)=u_{n}\left(t\right),\\ q=1\Rightarrow \varphi_{n}\left(t,1\right)=v_{n}\left(t\right). \end{array}$$ (8)

respectively. Thus, as *q* varies from 0 to 1, the solution *ϕ*_*n*_(*t*, *q*), *φ*_*n*_(*t*, *q*) varies from *u*_*n0*_(*t*), *v*_*n0*_(*t*) to *u*_*n*_(*t*), *v*_*n*_(*t*). Where *u*_*n0*_(*t*), *v*_*n0*_(*t*) are the zeroth order solutions which can be obtained from Eq 10.

Expanding *ϕ*_*n*_(*t*, *q*, *C*_*i*_), *φ*_*n*_(*t*, *q*, *C*_*i*_), *ϕ*_*n-k*_(*t*, *q*, *C*_*i*_), *φ*_*n-k*_(*t*, *q*, *C*_*i*_), *ϕ*_*n+k*_(*t*, *q*, *C*_*i*_), *φ*_*n+k*_(*t*, *q*, *C*_*i*_), by Taylors series and choosing *H*_1_(*q*), *H*_2_(*q*) as given

$$\begin{array}{l} H_{1}\left(q\right)=qC_{11}+q^{2}C_{12}+q^{3}C_{13}+\ldots +q^{m}C_{1m},\\ H_{2}\left(q\right)=qC_{21}+q^{2}C_{22}+q^{3}C_{23}+\ldots +q^{m}C_{2m},\\ \phi_{n-k}\left(t,q,C_{1i}\right)=u_{\left(n-k\right)0}\left(t\right)+\sum_{m=1}^{\infty }u_{\left(n-k\right)m}\left(t,C_{1i}\right)q^{m},\\ \varphi_{n-k}\left(t,q,C_{2i}\right)=v_{\left(n-k\right)0}\left(t\right)+\sum_{m=1}^{\infty }v_{\left(n-k\right)m}\left(t,C_{2i}\right)q^{m},\\ \phi_{n}\left(t,q,C_{1i}\right)=u_{\left(n\right)0}\left(t\right)+\sum_{m=1}^{\infty }u_{\left(n\right)m}\left(t,C_{1i}\right)q^{m},\\ \varphi_{n}\left(t,q,C_{2i}\right)=v_{\left(n\right)0}\left(t\right)+\sum_{m=1}^{\infty }v_{\left(n\right)m}\left(t,C_{2i}\right)q^{m},\\ \phi_{n+k}\left(t,q,C_{1i}\right)=u_{\left(n+k\right)0}\left(t\right)+\sum_{m=1}^{\infty }u_{\left(n+k\right)m}\left(t,C_{1i}\right)q^{m},\\ \varphi_{n+k}\left(t,q,C_{2i}\right)=v_{\left(n+k\right)0}\left(t\right)+\sum_{m=1}^{\infty }v_{\left(n+k\right)m}\left(t,C_{2i}\right)q^{m}, \end{array}$$ (9)

where *C*_1*i*_, *C*_2*i*_ *i =* 1, 2, 3, … are constants and to be determined and *m* ∈ *N*.

Now substituting Eq 9 into Eq 5 and equating the coefficient of like powers of *q*, we obtain

$$\begin{array}{l} 𝓛\left(u_{n0}(t)\right)+g\left(t\right)=0,\;𝓑\left(u_{n0}(t),\frac{\partial u_{n0}(t)}{\partial t}\right)=0\;,\\ 𝓛\left(v_{n0}(t)\right)+f\left(t\right)=0,\;𝓑\left(v_{n0}(t),\frac{\partial v_{n0}(t)}{\partial t}\right)=0\; \end{array}$$ (10)

$$\begin{array}{l} 𝓛\left(u_{n1}(t)\right)-𝓛\left(u_{n0}(t)\right)=C_{11}\left[𝓛\left(u_{n0}(t)\right)+𝓝\left(u_{\left(n-k\right)0}(t),u_{n0}(t),u_{\left(n+k\right)0}(t)\right)\right],\\ \;𝓑\left(u_{n1}(t),\frac{\partial u_{n1}(t)}{\partial t}\right)=0,\;\\ 𝓛\left(v_{n1}(t)\right)-𝓛\left(v_{n0}(t)\right)=C_{21}\left[𝓛\left(v_{n0}(t)\right)+𝓝\left(v_{\left(n-k\right)0}(t),v_{n0}(t),v_{\left(n+k\right)0}(t)\right)\right],\\ \;𝓑\left(v_{n1}(t),\frac{\partial v_{n1}(t)}{\partial t}\right)=0,\; \end{array}$$ (11)

$$\begin{array}{l} 𝓛\left(u_{n2}(t)\right)-𝓛\left(u_{n1}(t)\right)=C_{11}\left[𝓛\left(u_{n1}(t)\right)+𝓝\left(u_{\left(n-k\right)1}(t),u_{n1}(t),u_{\left(n+k\right)1}(t)\right)\right]\\ \;+C_{12}\left[𝓛\left(u_{n0}(t)\right)+𝓝\left(u_{\left(n-k\right)0}(t),u_{n0}(t),u_{\left(n+k\right)0}(t)\right)\right],\\ \;𝓑\left(u_{n2}(n,t),\frac{\partial u_{n2}(n,t)}{\partial t}\right)=0,\\ 𝓛\left(v_{n2}(t)\right)-𝓛\left(v_{n1}(t)\right)=C_{21}\left[𝓛\left(v_{n1}(t)\right)+𝓝\left(v_{\left(n-k\right)1}(t),v_{n1}(t),v_{\left(n+k\right)1}(t)\right)\right]\\ \;+C_{22}\left[𝓛\left(v_{n0}(t)\right)+𝓝\left(v_{\left(n-k\right)0}(t),v_{n0}(t),v_{\left(n+k\right)0}(t)\right)\right],\\ \;𝓑\left(v_{n2}(n,t),\frac{\partial v_{n2}(n,t)}{\partial t}\right)=0, \end{array}$$ (12)

$$\begin{array}{c} 𝓛\left(u_{n3}(t)\right)-𝓛\left(u_{n2}(t)\right)=C_{11}\left[𝓛\left(u_{n2}(t)\right)+𝓝\left(u_{\left(n-k\right)2}(t),u_{n2}(t),u_{\left(n+k\right)2}(t)\right)\right]\\ \;+C_{12}\left[𝓛\left(u_{n1}(t)\right)+𝓝\left(u_{\left(n-k\right)1}(t),u_{n1}(t),u_{\left(n+k\right)1}(t)\right)\right]\\ \;+C_{13}\left[𝓛\left(u_{n0}(t)\right)+𝓝\left(u_{\left(n-k\right)0}(t),u_{n0}(t),u_{\left(n+k\right)0}(t)\right)\right],\\ \;𝓑\left(u_{n3}(t),\frac{\partial u_{n3}(t)}{\partial t}\right)=0,\;\\ 𝓛\left(v_{n3}(t)\right)-𝓛\left(v_{n2}(t)\right)=C_{21}\left[𝓛\left(v_{n2}(t)\right)+𝓝\left(v_{\left(n-k\right)2}(t),v_{n2}(t),v_{\left(n+k\right)2}(t)\right)\right]\\ \;+C_{22}\left[𝓛\left(v_{n1}(t)\right)+𝓝\left(v_{\left(n-k\right)1}(t),v_{n1}(t),v_{\left(n+k\right)1}(t)\right)\right]\\ \;+C_{23}\left[𝓛\left(v_{n0}(t)\right)+𝓝\left(v_{\left(n-k\right)0}(t),v_{n0}(t),v_{\left(n+k\right)0}(t)\right)\right],\\ \;𝓑\left(v_{n3}(t),\frac{\partial v_{n3}(t)}{\partial t}\right)=0,\;\\ \vdots \end{array}$$ (13)

$$\begin{array}{l} 𝓛\left(u_{n\;j}(t)\right)-𝓛\left(u_{n\;\left(j-1\right)}(t)\right)=\sum_{i=1}^{m}\sum_{j=m-1}^{0}C_{1i}\left[𝓛\left(u_{n\;j}(t)\right)+𝓝\left(u_{n\;j}(t),u_{\left(n-k\right)\;j}(t),u_{\left(n+k\right)\;j}(t)\right)\right]\\ \;𝓑\left(u_{n\;j}(t),\frac{\partial u_{n\;j}(t)}{\partial t}\right)=0,\\ 𝓛\left(v_{n\;j}(t)\right)-𝓛\left(v_{n\;\left(j-1\right)}(t)\right)=\sum_{i=1}^{m}\sum_{j=m-1}^{0}C_{2i}\left[𝓛\left(v_{n\;j}(t)\right)+𝓝\left(v_{n\;j}(t),v_{\left(n-k\right)\;j}(t),v_{\left(n+k\right)\;j}(t)\right)\right]\\ \;𝓑\left(v_{n\;j}(t),\frac{\partial v_{n\;j}(t)}{\partial t}\right)=0. \end{array}$$ (14)

We obtained the zeroth order solution, first, second, third and the general order solutions by solving Eqs 10–14. In the same way the remaining solutions can be determined. It has been observed that the convergence of the series (9) depends upon the auxiliary constants *C*_1*i*_, *C*_2*i*_. If it is convergent at *q* = 1, one has

$$\begin{array}{c} u_{n}\left(t;C_{1i}\right)=\;u_{n}{}_{0}(x,t)\;+\sum_{k\geq 1}u_{n}{}_{k}\left(t;C_{1i}\right),\\ v_{n}\left(t;C_{2i}\right)=\;v_{n}{}_{0}(x,t)\;+\sum_{k\geq 1}v_{n}{}_{k}\left(t;C_{2i}\right). \end{array}$$ (15)

Substituting Eq 15 into Eq 1, it results the following expression for residual

$$\begin{array}{c} R_{1}\left(t,C_{1i}\right)=𝓛({\tilde{u}}_{n}(t,C_{1i}))+g(t)+𝓝({\tilde{u}}_{n}(t,C_{1i})),\\ R_{2}\left(t,C_{2i}\right)=𝓛({\tilde{v}}_{n}(t,C_{2i}))+f(t)+𝓝({\tilde{v}}_{n}(t,C_{2i})). \end{array}$$ (16)

If *R*_1_(*t*, *C*_*1i*_) = 0, *R*_*2*_(*t*, *C*_*2i*_) = 0 then${\tilde{u}}_{n}(t,C_{1i})$, $\;{\tilde{v}}_{n}\left(t,C_{2i}\right)$ is the exact solution of the problem. Generally it doesn’t happen, especially in nonlinear problems.

For the determinations of auxiliary constants, *C*_*1i*_, *C*_*2i*_ *i =* 1, 2, 3,…, there are different methods like Galerkin’s Method, Ritz Method, Least Squares Method and Collocation Method. One can apply the Method of Least Squares as under

$$\begin{array}{l} J_{1}\left(C_{1i}\right)=\int_{a}^{b}\;\int_{\Omega }^{}R_{1}{}^{2}\left(t,C_{1i}\right)\;dndt,\\ J_{2}\left(C_{2i}\right)=\int_{c}^{d}\;\int_{\Xi }^{}R_{2}{}^{2}\left(t,C_{2i}\right)\;dndt, \end{array}$$ (17)

where *a*, *b*, *c*, *d* are four values, depending on the nature of the given problem.

The auxiliary constants *C*_*1i*_, *C*_*2i*_ can be optimally calculated as

$$\begin{array}{l} \frac{\partial J_{1}}{\partial C_{11}}=\frac{\partial J_{1}}{\partial C_{12}}=\frac{\partial J_{1}}{\partial C_{13}}\ldots \frac{\partial J_{1}}{\partial C_{1m}}=0,\\ \frac{\partial J_{2}}{\partial C_{21}}=\frac{\partial J_{2}}{\partial C_{22}}=\frac{\partial J_{2}}{\partial C_{23}}\ldots \frac{\partial J_{2}}{\partial C_{2m}}=0, \end{array}$$ (18)

The *m*th order approximate solution can be obtained by these constants.

The convergence of OHAM is directly proportional to the number of optimal constants *C*_*1i*_, *C*_*2i*_.

## Application of Modified OHAM to Coupled Differential-Difference Equations

To show the validity and effectiveness of modified OHAM formulation to coupled differential-difference equation, we consider Relativistic Toda coupled nonlinear differential-difference equation of the form:

$$\begin{array}{l} \frac{\partial u_{n}\left(t\right)}{\partial t}=\left(1+\beta u_{n}\left(t\right)\right)\left(v_{n}\left(t\right)-v_{n-1}\left(t\right)\right),\\ \frac{\partial v_{n}\left(t\right)}{\partial t}=v_{n}\left(t\right)\left(u_{n+1}\left(t\right)-u_{n}\left(t\right)+\beta v_{n+1}\left(t\right)-\beta v_{n-1}\left(t\right)\right), \end{array}$$ (19)

with initial conditions

$$\begin{array}{l} u_{n}\left(0\right)=-1-a\coth\left(b\right)+a\tanh\left(bn\right),\\ v_{n}\left(0\right)=a\coth\left(b\right)-a\tanh\left(bn\right). \end{array}$$ (20)

we take,

$$\begin{array}{l} u_{n}\left(t\right)=u_{n0}\left(t\right)+pu_{n1}\left(t\right)+p^{2}u_{n2}\left(t\right)+p^{3}u_{n3}\left(t\right),\\ u_{n+1}\left(t\right)=u_{\left(n+1\right)0}\left(t\right)+pu_{\left(n+1\right)1}\left(t\right)+p^{2}u_{\left(n+1\right)2}\left(t\right)+p^{3}u_{\left(n+1\right)3}\left(t\right),\\ u_{n-1}\left(t\right)=u_{\left(n-1\right)0}\left(t\right)+pu_{\left(n-1\right)1}\left(t\right)+p^{2}u_{\left(n-1\right)2}\left(t\right)+p^{3}u_{\left(n-1\right)3}\left(t\right),\\ v_{n}\left(t\right)=v_{n0}\left(t\right)+pv_{n1}\left(t\right)+p^{2}v_{n2}\left(t\right)+p^{3}v_{n3}\left(t\right),\\ v_{n+1}\left(t\right)=v_{\left(n+1\right)0}\left(t\right)+pv_{\left(n+1\right)1}\left(t\right)+p^{2}v_{\left(n+1\right)2}\left(t\right)+p^{3}v_{\left(n+1\right)3}\left(t\right),\\ v_{n-1}\left(t\right)=v_{\left(n-1\right)0}\left(t\right)+pv_{\left(n-1\right)1}\left(t\right)+p^{2}v_{\left(n-1\right)2}\left(t\right)+p^{3}v_{\left(n-1\right)3}\left(t\right). \end{array}$$ (21)

According to Eq 1 we have

$$\begin{array}{l} {𝓛}_{1}=\frac{\partial u_{n}\left(t\right)}{\partial t},\;f\left(t\right)=0,\;{𝓝}_{1}=-\left(1+\beta u_{n}\left(t\right)\right)\left(v_{n}\left(t\right)-v_{n-1}\left(t\right)\right),\\ {𝓛}_{2}=\frac{\partial v_{n}\left(t\right)}{\partial t},\;g\left(t\right)=0,\;{𝓝}_{2}=-v_{n}\left(t\right)\left(u_{n+1}\left(t\right)-u_{n}\left(t\right)+\beta v_{n+1}\left(t\right)-\beta v_{n-1}\left(t\right)\right), \end{array}$$ (22)

Using Eqs 19, 20 and 21 into Eq 5 and using the method discussed in section “Application of Modified OHAM to Coupled Differential-Difference Equations” leads to the following.

### Zeroth Order System

$$\begin{array}{l} \frac{\partial u_{n0}\left(t\right)}{\partial t}=0,\\ \frac{\partial v_{n0}\left(t\right)}{\partial t}=0, \end{array}$$ (23)

with

$$\begin{array}{l} u_{n0}\left(0\right)=-1-a\coth\left(b\right)+a\tanh\left(bn\right),\\ v_{n0}\left(0\right)=a\coth\left(b\right)-a\tanh\left(bn\right), \end{array}$$ (24)

from which we obtain

$$\begin{array}{l} u_{n0}\left(t\right)=-1-a\coth\left(b\right)+a\tanh\left(bn\right),\\ v_{n0}\left(t\right)=a\coth\left(b\right)-a\tanh\left(bn\right). \end{array}$$ (25)

### First Order System

$$\begin{array}{l} \frac{\partial u_{n1}\left(t\right)}{\partial t}=\left(1+C_{11}\right)\frac{\partial u_{n0}\left(t\right)}{\partial t}+C_{11}\left(1+\beta u_{n0}\right)v_{\left(n-1\right)0}\left(t\right)-C_{11}\left(1+\beta u_{n0}\right)v_{n0}\left(t\right),\\ \frac{\partial v_{n1}\left(t\right)}{\partial t}=\left(1+C_{21}\right)\frac{\partial v_{n0}\left(t\right)}{\partial t}+\beta C_{21}\left(1+\beta u_{n0}\right)\left(v_{\left(n-1\right)0}\left(t\right)-v_{\left(n+1\right)0}\left(t\right)\right)v_{n0}\left(t\right)\\ \;-C_{21}\left(u_{\left(n+1\right)0}\left(t\right)-u_{n0}\left(t\right)\right)v_{n0}\left(t\right), \end{array}$$ (26)

with

$$\begin{array}{l} u_{n1}\left(0\right)=0,\\ v_{n1}\left(0\right)=0, \end{array}$$ (27)

its solution is

$$\begin{array}{l} u_{n1}\left(t\right)=C_{11}t\left[\left(\tanh\left(bn\right)-\tanh\left(b\left(n-1\right)\right)\right)\left(\begin{array}{l} \left(1-\beta \right)a-a^{2}\beta \coth\left(b\right)\\ +a^{2}\beta \tanh\left(bn\right) \end{array}\right)\right],\\ v_{n1}\left(t\right)=-C_{21}t\left[\begin{array}{l} a^{2}\beta \coth\left(b\right)\tanh\left(b\left(n-1\right)\right)-a^{2}\coth\left(b\right)\tanh\left(bn\right)-\\ a^{2}\beta \tanh\left(bn\right)\tanh\left(b\left(n-1\right)\right)+a^{2}\tanh^{2}\left(bn\right)+\\ a^{2}\coth\left(b\right)\tanh\left(b\left(n+1\right)\right)-a^{2}\beta \coth\left(b\right)\tanh\left(b\left(n+1\right)\right)\\ -a^{2}\tanh\left(bn\right)\tanh\left(b\left(n+1\right)\right)+a^{2}\beta \tanh\left(bn\right)\tanh\left(b\left(n+1\right)\right) \end{array}\right]. \end{array}$$ (28)

### Second Order System

$$\begin{array}{l} \frac{\partial u_{n2}\left(t\right)}{\partial t}=\left(1+C_{11}\right)\frac{\partial u_{n1}\left(t\right)}{\partial t}+C_{12}\frac{\partial u_{n0}\left(t\right)}{\partial t}-C_{11}\left(1+\beta u_{n0}\left(t\right)\right)v_{n1}\left(t\right)\\ -\beta C_{11}u_{n1}\left(t\right)v_{n0}\left(t\right)-C_{12}\left(1+\beta u_{n0}\left(t\right)\right)v_{n0}\left(t\right)+\beta C_{11}u_{n1}\left(t\right)v_{\left(n-1\right)0}\left(t\right)\\ +C_{12}\left(1+\beta u_{n0}\left(t\right)\right)v_{\left(n-1\right)0}\left(t\right)+C_{11}\left(1+\beta u_{n0}\left(t\right)\right)v_{\left(n-1\right)1}\left(t\right),\\ \frac{\partial v_{n2}\left(t\right)}{\partial t}=\left(1+C_{21}\right)\frac{\partial v_{n1}\left(t\right)}{\partial t}+C_{22}\frac{\partial v_{n0}\left(t\right)}{\partial t}+\beta C_{21}\left(v_{\left(n-1\right)0}\left(t\right)-v_{\left(n+1\right)0}\left(t\right)\right)v_{n1}\left(t\right)+\\ \beta C_{21}\left(v_{\left(n-1\right)1}\left(t\right)-v_{\left(n+1\right)1}\left(t\right)\right)v_{n0}\left(t\right)+\beta C_{22}\left(v_{\left(n-1\right)0}\left(t\right)-v_{\left(n+1\right)0}\left(t\right)\right)v_{n0}\left(t\right)\\ +C_{22}\left(u_{n0}\left(t\right)-u_{\left(n+1\right)0}\left(t\right)\right)v_{n0}\left(t\right)+C_{21}\left(u_{n1}\left(t\right)-u_{\left(n+1\right)1}\left(t\right)\right)v_{n0}\left(t\right)+\\ C_{21}u_{n0}\left(t\right)v_{n0}\left(t\right)-C_{21}u_{\left(n+1\right)0}\left(t\right)v_{n1}\left(t\right), \end{array}$$ (29)

with

$$\begin{array}{l} u_{n2}\left(0\right)=0,\\ v_{n2}\left(0\right)=0, \end{array}$$ (30)

its solution is

$$\begin{array}{l} u_{n2}\left(t\right)=-\frac{1}{2}at\left(-1+\beta a\coth\left(b\right)-a\beta \tanh\left(bn\right)\right)\times \\ \;\left[\begin{array}{l} (\left(2C_{12}-C_{11}^{2}\right)\left(\tanh\left(bn\right)-\tanh\left(b\left(n-1\right)\right)\right)(-2+at\beta \left(\tanh\left(bn\right)-\tanh\left(b\left(n-1\right)\right)\right)\\ +C_{11}\left(-2\tanh\left(b\left(n-1\right)\right)+2\tanh\left(bn\right)\right)+atC_{21}(\beta \tanh\left(b\left(n-2\right)\right)\tanh\left(b\left(n-1\right)\right)\\ -\tanh^{2}\left(b\left(n-1\right)\right)+\tanh\left(b\left(n-1\right)\right)\tanh\left(bn\right)-2\beta \tanh\left(b\left(n-1\right)\right)\tanh\left(bn\right)\\ +\tanh^{2}\left(bn\right)-\tanh\left(bn\right)\tanh\left(b\left(n+1\right)\right)+\beta \tanh\left(bn\right)\tanh\left(b\left(n+1\right)\right)+\\ \coth\left(b\right)(-\beta \tanh\left(b\left(n-2\right)\right)+(1+\beta )\tanh\left(b\left(n-1\right)\right)-2\tanh\left(bn\right)+\\ \beta \tanh\left(bn\right)+\tanh\left(b\left(n+1\right)\right)-\beta \tanh\left(b\left(n+1\right)\right)))) \end{array}\right].\\ v_{n2}\left(t\right)=\frac{1}{2}at^{2}(\coth\left(b\right)-\tanh\left(bn\right)\times \\ \;\left[\begin{array}{l} (2C_{22}(-\beta \tanh\left(b\left(n-1\right)\right)+\tanh\left(bn\right)+\\ \left(\beta -1\right)\tanh\left(b\left(n+1\right)\right)+C_{21}(2\;(-\beta \tanh\left(b\left(n-1\right)\right)+\tanh\left(bn\right)+\\ \left(\beta -1\right)\tanh\left(b\left(n+1\right)\right)+tC_{11}(\beta a\tanh^{2}\left(bn\right)+\tanh\left(b\left(n-1\right)\right)(-1\\ +\beta +a\beta \coth\left(b\right)-a\beta \tanh\left(bn\right))+\tanh\left(b\left(n+1\right)\right)(-1+\beta +\\ a\beta \coth\left(b\right)-a\beta \tanh\left(b\left(n+1\right)\right)+\tanh\left(bn\right)(2-2\beta -2a\beta \coth\left(b\right)\\ +a\beta \tanh\left(b\left(n+1\right)\right)))-C_{21}^{2}(-at\left(\beta -1\right)\beta \tanh^{2}\left(b\left(n-1\right)\right)-2\tanh\left(bn\right)\\ -\;at\tanh^{2}\left(bn\right)+2\tanh\left(b\left(n+1\right)\right)-2\beta \tanh\left(b\left(n+1\right)\right)+\\ 2at\tanh\left(bn\right)\tanh\left(b\left(n+1\right)\right)-2at\beta \tanh\left(bn\right)\tanh\left(b\left(n+1\right)\right)\\ +at\beta^{2}\tanh\left(bn\right)\tanh\left(b\left(n+1\right)\right)-at\tanh^{2}\left(b\left(n+1\right)\right)+at\beta \tanh^{2}\\ \left(b\left(n+1\right)\right)-at\beta^{2}\tanh^{2}\left(b\left(n+1\right)\right)+\beta \tanh\left(b\left(n-1\right)\right)(2-at\beta \tanh\\ \left(b\left(n-2\right)\right)+at\left(1+\beta \right)\tanh\left(bn\right)-2at\tanh\left(b\left(n+1\right)\right)+2at\beta \tanh\left(b\left(n+1\right)\right)\\ +at\beta \tanh\left(b\left(n+1\right)\right)\tanh\left(b\left(n+2\right)\right)-at\beta^{2}\tanh\left(b\left(n+1\right)\right)\tanh\left(b\left(n+2\right)\right)\\ +at\beta \coth\left(b\right)(\beta \tanh\left(b\left(n-2\right)\right)-\tanh\left(b\left(n-1\right)\right)+\tanh\left(bn\right)-2\beta \tanh\left(bn\right)\\ +\tanh\left(b\left(n+1\right)\right)-\tanh\left(b\left(n+2\right)\right)+\beta \tanh\left(b\left(n+2\right)\right))). \end{array}\right] \end{array}$$ (31)

Adding Eqs 25, 28, and 31, we obtain

$$\begin{array}{l} u_{n}\left(t,C_{11},C_{12}\right)=u_{n0}\left(t\right)+u_{n1}\left(t,C_{11}\right)+u_{n2}\left(t,C_{11},C_{12}\right),\\ v_{n}\left(t,C_{21},C_{22}\right)=v_{n0}\left(t\right)+v_{n1}\left(t,C_{21}\right)+v_{n2}\left(t,C_{21},C_{22}\right), \end{array}$$ (32)

$$\begin{array}{l} u_{n}\left(t\right)=-1-a\coth\left(b\right)+a\tanh\left(bn\right)+\\ \;C_{11}t\left[\left(\tanh\left(bn\right)-\tanh\left(b\left(n-1\right)\right)\right)\left(\begin{array}{l} \left(1-\beta \right)a-a^{2}\beta \coth\left(b\right)\\ +a^{2}\beta \tanh\left(bn\right) \end{array}\right)\right]+\;\\ -\frac{1}{2}at\left(-1+\beta a\coth\left(b\right)-a\beta \tanh\left(bn\right)\right)\times \\ \;\left[\begin{array}{l} (\left(2C_{12}-C_{11}^{2}\right)\left(\tanh\left(bn\right)-\tanh\left(b\left(n-1\right)\right)\right)(-2+at\beta \left(\tanh\left(bn\right)-\tanh\left(b\left(n-1\right)\right)\right)\\ +C_{11}\left(-2\tanh\left(b\left(n-1\right)\right)+2\tanh\left(bn\right)\right)+atC_{21}(\beta \tanh\left(b\left(n-2\right)\right)\tanh\left(b\left(n-1\right)\right)\\ -\tanh^{2}\left(b\left(n-1\right)\right)+\tanh\left(b\left(n-1\right)\right)\tanh\left(bn\right)-2\beta \tanh\left(b\left(n-1\right)\right)\tanh\left(bn\right)\\ +\tanh^{2}\left(bn\right)-\tanh\left(bn\right)\tanh\left(b\left(n+1\right)\right)+\beta \tanh\left(bn\right)\tanh\left(b\left(n+1\right)\right)+\\ \coth\left(b\right)(-\beta \tanh\left(b\left(n-2\right)\right)+(1+\beta )\tanh\left(b\left(n-1\right)\right)-2\tanh\left(bn\right)+\\ \beta \tanh\left(bn\right)+\tanh\left(b\left(n+1\right)\right)-\beta \tanh\left(b\left(n+1\right)\right)))) \end{array}\right].\\ v_{n}\left(t\right)=a\coth\left(b\right)-a\tanh\left(bn\right)\\ -C_{21}t\left[\begin{array}{l} a^{2}\beta \coth\left(b\right)\tanh\left(b\left(n-1\right)\right)-a^{2}\coth\left(b\right)\tanh\left(bn\right)-\\ a^{2}\beta \tanh\left(bn\right)\tanh\left(b\left(n-1\right)\right)+a^{2}\tanh^{2}\left(bn\right)+\\ a^{2}\coth\left(b\right)\tanh\left(b\left(n+1\right)\right)-a^{2}\beta \coth\left(b\right)\tanh\left(b\left(n+1\right)\right)\\ -a^{2}\tanh\left(bn\right)\tanh\left(b\left(n+1\right)\right)+a^{2}\beta \tanh\left(bn\right)\tanh\left(b\left(n+1\right)\right) \end{array}\right]\\ \frac{1}{2}at^{2}(\coth\left(b\right)-\tanh\left(bn\right)\times \\ \;\left[\begin{array}{l} (2C_{22}(-\beta \tanh\left(b\left(n-1\right)\right)+\tanh\left(bn\right)+\\ \left(\beta -1\right)\tanh\left(b\left(n+1\right)\right)+C_{21}(2\;(-\beta \tanh\left(b\left(n-1\right)\right)+\tanh\left(bn\right)+\\ \left(\beta -1\right)\tanh\left(b\left(n+1\right)\right)+tC_{11}(\beta a\tanh^{2}\left(bn\right)+\tanh\left(b\left(n-1\right)\right)(-1\\ +\beta +a\beta \coth\left(b\right)-a\beta \tanh\left(bn\right))+\tanh\left(b\left(n+1\right)\right)(-1+\beta +\\ a\beta \coth\left(b\right)-a\beta \tanh\left(b\left(n+1\right)\right)+\tanh\left(bn\right)(2-2\beta -2a\beta \coth\left(b\right)\\ +a\beta \tanh\left(b\left(n+1\right)\right)))-C_{21}^{2}(-at\left(\beta -1\right)\beta \tanh^{2}\left(b\left(n-1\right)\right)-2\tanh\left(bn\right)\\ -\;at\tanh^{2}\left(bn\right)+2\tanh\left(b\left(n+1\right)\right)-2\beta \tanh\left(b\left(n+1\right)\right)+\\ 2at\tanh\left(bn\right)\tanh\left(b\left(n+1\right)\right)-2at\beta \tanh\left(bn\right)\tanh\left(b\left(n+1\right)\right)\\ +at\beta^{2}\tanh\left(bn\right)\tanh\left(b\left(n+1\right)\right)-at\tanh^{2}\left(b\left(n+1\right)\right)+at\beta \tanh^{2}\\ \left(b\left(n+1\right)\right)-at\beta^{2}\tanh^{2}\left(b\left(n+1\right)\right)+\beta \tanh\left(b\left(n-1\right)\right)(2-at\beta \tanh\\ \left(b\left(n-2\right)\right)+at\left(1+\beta \right)\tanh\left(bn\right)-2at\tanh\left(b\left(n+1\right)\right)+2at\beta \tanh\left(b\left(n+1\right)\right)\\ +at\beta \tanh\left(b\left(n+1\right)\right)\tanh\left(b\left(n+2\right)\right)-at\beta^{2}\tanh\left(b\left(n+1\right)\right)\tanh\left(b\left(n+2\right)\right)\\ +at\beta \coth\left(b\right)(\beta \tanh\left(b\left(n-2\right)\right)-\tanh\left(b\left(n-1\right)\right)+\tanh\left(bn\right)-2\beta \tanh\left(bn\right)\\ +\tanh\left(b\left(n+1\right)\right)-\tanh\left(b\left(n+2\right)\right)+\beta \tanh\left(b\left(n+2\right)\right))). \end{array}\right] \end{array}$$ (33)

For the computation of the constants *C*_11_, *C*_12_, *C*_21_ and *C*_22_ applying the method of least square mentioned in Eqs 16–18, we get

*C*_11_ = −1.5137123813420987, *C*_12_ = −4.052215660646143

*C*_21_ = −1.1471315280070562, *C*_22_ = −0.6627903580361686

for *n* = 1, *a* = 1, *b* = 1, *β* = 0.1.

Putting these values in Eq 33, we obtained the approximate solution of the form

$$\begin{array}{l} u_{n}\left(t\right)=\left[\begin{array}{l} -1-\coth\left(1\right)+\tanh\left(n\right)+t(-1.16359\tanh\left(1-n\right)-1.16359\tanh\left(n\right)-\\ 0.151371\tanh\left(1-n\right)\tanh\left(n\right)-0.151371\tanh^{2}\left(n\right))+\frac{1}{2}(-5.03435t\\ \tanh\left(1-n\right)-1.92788t^{2}\tanh\left(1-n\right)-1.15865t^{2}\tanh^{2}\left(1-n\right)+\\ 0.175262t^{2}\tanh\left(2-n\right)+0.133479t^{2}\tanh\left(1-n\right)\tanh\left(2-n\right)-\\ 5.03435t\tanh\left(n\right)-3.32998t^{2}\tanh\left(n\right)-0.654921t\tanh\left(1-n\right)\tanh\left(n\right)\\ -0.966361t^{2}\tanh\left(1-n\right)\tanh\left(n\right)-0.150729t^{2}\tanh^{2}\left(1-n\right)\tanh\left(n\right)+\\ 0.01373643t^{2}\tanh\left(1-n\right)\tanh\left(2-n\right)\tanh\left(n\right)-0.654921t\tanh^{2}\left(n\right)+\\ 1.07772t^{2}\tanh^{2}\left(n\right)-0.0930877t^{2}\tanh\left(1-n\right)\tanh^{2}\left(n\right)+0.196556t^{2}\\ \tanh^{3}\left(n\right)+1.57736t^{2}\tanh\left(1+n\right)-0.996108t^{2}\tanh\left(n\right)\tanh\left(1+n\right)-\\ 0.156278t^{2}\tanh^{2}\left(n\right)\tanh\left(1+n\right) \end{array}\right]\\ v_{n}\left(t\right)=\left[\begin{array}{l} \coth\left(1\right)-\tanh\left(n\right)-t(0.150622\tanh\left(1-n\right)+1.50622\tanh\left(n\right)-0.114713\tanh\left(1-n\right)\tanh\left(n\right)\\ -1.14713\tanh^{2}\left(n\right)-1.3556\tanh\left(1+n\right)+1.03242\tanh\left(n\right)\tanh\left(1+n\right))+\frac{1}{2}(-0.129731t\\ \tanh\left(1-n\right)+1.52575t^{2}\tanh\left(1-n\right)-0.155505t^{2}\tanh^{2}\left(1-n\right)+0.0226871t^{2}\tanh\left(2-n\right)+\\ 0.0172784t^{2}\tanh\left(1-n\right)\tanh\left(2-n\right)-1.29731t\tanh\left(n\right)+3.32374t^{2}\tanh\left(n\right)+0.0988022t\\ \tanh\left(1-n\right)\tanh\left(n\right)-0.743941t^{2}\tanh\left(1-n\right)\tanh\left(n\right)+0.118432t^{2}\tanh^{2}\left(1-n\right)\tanh\left(n\right)+\\ 0.0172784t^{2}\tanh\left(2-n\right)\tanh\left(n\right)-0.0131591t^{2}\tanh\left(1-n\right)\tanh\left(2-n\right)\tanh\left(n\right)+0.988022\\ t\tanh^{2}\left(n\right)-0.575508t^{2}\tanh^{2}\left(n\right)-0.318393t^{2}\tanh\left(1-n\right)\tanh^{2}\left(n\right)-1.48955t^{2}\tanh^{3}\left(n\right)+\\ 1.57736t^{2}\tanh\left(1+n\right)-0.996108t^{2}\tanh\left(n\right)\tanh\left(1+n\right)-\\ 1.16758\tanh\left(1+n\right)-1.97949t^{2}\tanh\left(1+n\right)-0.311011t^{2}\tanh\left(1-n\right)\tanh\left(1+n\right)-0.88922\\ t\tanh\left(n\right)\tanh\left(1+n\right)-1.39182t^{2}\tanh\left(n\right)\tanh\left(1+n\right)+0.236864t^{2}\tanh\left(1-n\right)\tanh\left(n\right)\\ \tanh\left(1+n\right)+2.20816t^{2}\tanh^{2}\left(n\right)\tanh\left(1+n\right)+1.34433t^{2}\tanh^{2}\left(1+n\right)-1.02384t^{2}\tanh\left(n\right)\\ \tanh^{2}\left(1+n\right)+0.204184t^{2}\tanh\left(2+n\right)-0.155505t^{2}\tanh\left(n\right)\tanh\left(2+n\right)-0.155505t^{2}\\ \tanh\left(1+n\right)\tanh\left(2+n\right)+0.118432t^{2}\tanh\left(n\right)\tanh\left(1+n\right)\tanh\left(2+n\right)) \end{array}\right] \end{array}$$ (34)

The Adomian Solution of Eq 19 is given by [12]

$$\begin{array}{l} u_{n}\left(t\right)=-1-a\coth\left(b\right)+a\tanh\left(bn+at\right),\\ v_{n}\left(t\right)=a\coth\left(b\right)-a\tanh\left(bn+at\right), \end{array}$$ (35)

where *a* and *b* are constants.

## Results and Discussion

**Fig 1:**
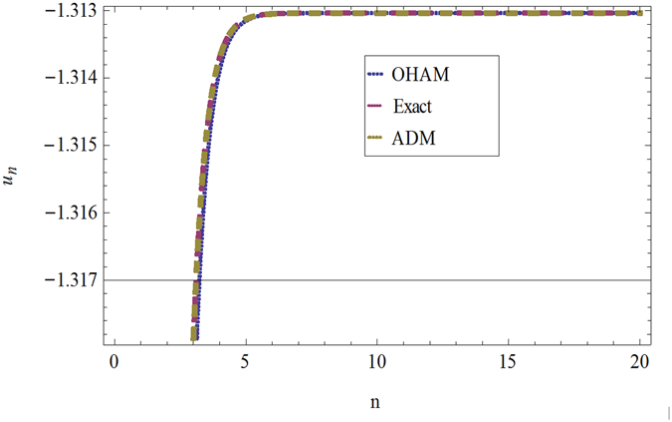
Comparison of OHAM, Exact and ADM solutions for *u*_*n*_(*t*), when *a* = 1, *b* = 1, *β* = 0.1 and *t* = .01.

**Fig 2:**
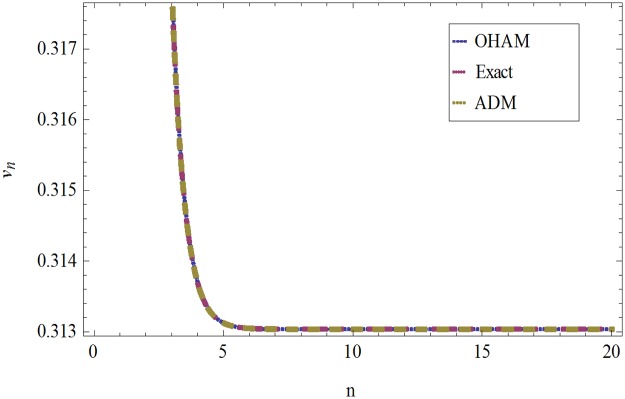
Comparison of OHAM, Exact and ADM solutions for *ν*_*n*_(*t*) when *a* = 1, *b* = 1, *β* = 0.1and *t* = .01.

**Fig 3:**
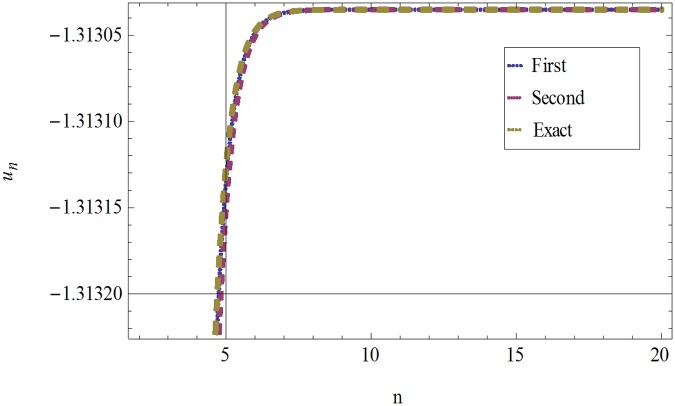
Comparison of First, Second and Exact solutions for *u*_*n*_(*t*) when *a* = 1, *b* = 1, *β* = 0.1and *t* = .01.

**Fig 4:**
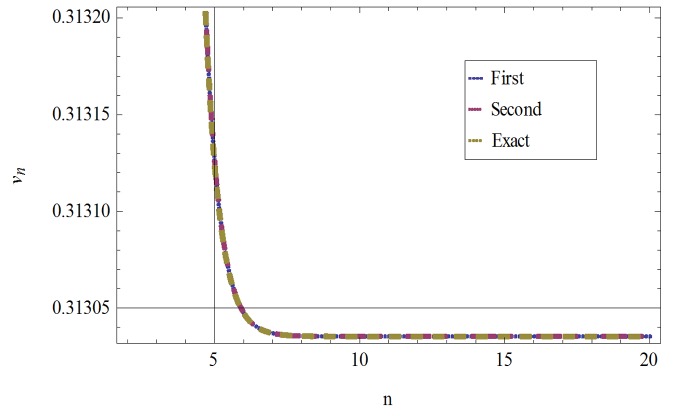
Comparison of First, Second, Third and Exact solutions for *ν*_*n*_(*t*) when *a* = 1, *b* = 1, *β* = 0.1and *t* = .01.

**Fig 5:**
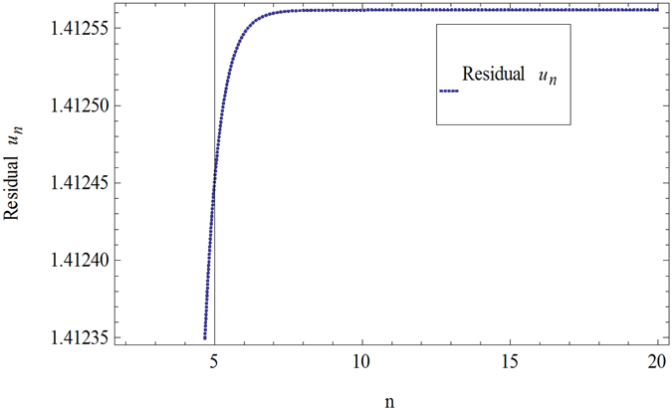
Residual of Eq 19 for *u*_*n*_(*t*) when *a* = 1, *b* = 1, *β* = 0.1and *t* = .01.

**Fig 6:**
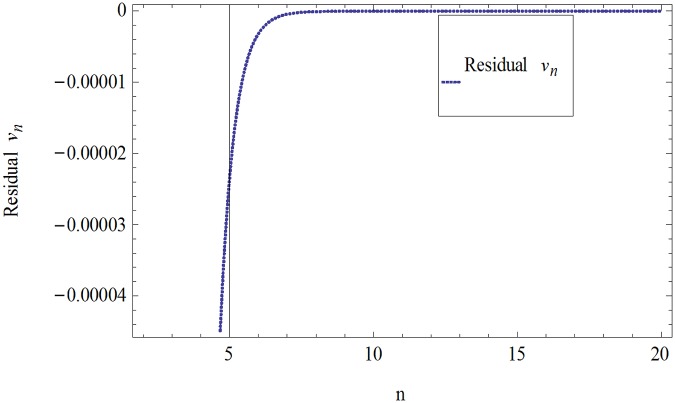
Residual of Eq 19 for *ν*_*n*_(*t*) when *a* = 1, *b* = 1, *β* = 0.1and *t* = .01.

The formulation presented in section 2, provides highly accurate solutions for the problems demonstrated in section 3. We have used Mathematica 7 for most of our computational work. Tables 1 and 2 and Figs 1 and 2 give the comparisons of OHAM with ADM and exact solutions. Also the absolute errors at different values of *n* at *t* = 5.1and *a* = 1, *b* = 1, *β* = 0.1 are given. The convergence of approximate orders to exact solutions are given in Figs 3 and 4 for *u*_*n*_(*t*) and *ν*_*n*_(*t*) respectively for *a* = 1, *b* = 1, *β* = 0.1 and *t* = .01. The residuals have been plotted in Figs 5 and 6 for *u*_*n*_(*t*) and *ν*_*n*_(*t*) at *t* = .01 and *a* = 1, *b* = 1, *β* = 0.1. We have concluded that the results obtained by OHAM are strongly identical to the results obtained by ADM and Exact. OHAM converge rapidly with increasing the order of approximation.

**Table 1:** Comparisons of OHAM, Exact and ADM Results of *u*_*n*_(*t*) at different values of *n* at *t* = 5.1.

*n*	OHAM	Exact [26]	ADM	E*
4	-1.408393259489	-1.387212348278	-1.313035310437	0.02118091
6	-1.314780295101	-1.314770184211	-1.313035285956	1.704596×10^–3^
8	-1.313067245943	-1.313045245721	-1.313035285507	2.200022×10^–5^
10	-1.313035870875	-1.313035616254	-1.313035285499	2.546210×10^–7^
12	-1.313035296220	-1.313035294882	-1.313035285499	1.338000×10^–9^
14	-1.313035285695	-1.313035285693	-1.313035285499	1.999956×10^–12^
16	-1.313035285499	-1.313035285499	-1.313035285499	0.00000000000
18	-1.313035285499	-1.313035825499	-1.313035285499	0.00000000000
20	-1.313035285499	-1.313035285499	-1.313035285499	0.00000000000

E* = |*OHAM*—*Exact*|

**Table 2:** Comparisons of OHAM, Exact and ADM Results of *v*_*n*_(*t*) at different values of *n* at *t* = 5.1.

*n*	OHAM	Exact [26]	ADM	E*
4	0.336563955725	0.325363854214	0.313035104378	0.0112001
6	0.313466842974	0.313355731862	0.313035285956	0.0001111
8	0.313043189956	0.313033178843	0.313035285507	1.91239×10^–5^
10	0.313035430274	0.313035331187	0.313035285499	4.96780×10^–8^
12	0.313035281509	0.313035281507	0.313035285499	2.00001×10^–12^
14	0.313035285547	0.313035285546	0.313035285499	9.99977×10^–13^
16	0.313035174499	0.313035174499	0.313035285499	0.0000000000
18	0.313035285499	0.313035285499	0.313035285499	0.0000000000
20	0.313035185499	0.313035285499	0.313035285499	0.0000000000

## Conclusion

The results obtained reveal the usefulness of OHAM. The OHAM is very efficient and powerful method in obtaining the solutions of the nonlinear coupled differential-difference equations. It is clear that this method does not require linearization and unrealistic assumptions and presents efficient numerical solutions. We have concluded from numerical results that OHAM provides very accurate results when it is compared with other method such as ADM and Exact solutions. We found it simpler in applicability, more convenient to control convergence and involved less computational overhead. The results obtained by OHAM are identical to the results obtained by ADM and Exact proving its validity and great potential for the solutions of DDEs. In this work, we have seen the effectiveness of OHAM [12–16] to DDEs. By applying the basic idea of OHAM to differential-difference equations, we found it simpler in applicability, more convenient to control convergence and involved less computational overhead. Therefore, OHAM shows its validity and great potential for the differential-difference equations arising in science and engineering.
